# Production and evaluation of mineral and nutrient contents, chemical composition, and sensory properties of ice creams fortified with laboratory-prepared peach fibre

**DOI:** 10.3402/fnr.v60.31882

**Published:** 2016-11-03

**Authors:** Filiz Yangılar

**Affiliations:** Department of Nutrition and Dietetics, Faculty of Health Sciences, Erzincan University, Erzincan, Turkey

**Keywords:** ice cream, peach fibre, chemical composition

## Abstract

**Background:**

In the coming years, a nutraceutical food may provide both physical and mental benefits that are commonly attributed to the active components of the food.

**Objective:**

In this study, we determined the nutrient and mineral contents, sensory properties, and physical and chemical characteristics of ice creams manufactured using peach fibre at different concentrations (1 and 2%).

**Method:**

A total of five experimental groups were formed: two types (from peach peel and pulp) of flour, two fibre concentrations (1 and 2%), and a control group without fibres.

**Results:**

Flour obtained from peach pulp and peel was found to have a significant (*p*<0.05) effect on the chemical composition and elemental composition of ice cream samples, especially the rates of Ca, K, Mg, and P, which increased in the samples depending on the content of peach fibre. Sensory ratings and acceptability of ice creams decreased significantly with increasing peach peel fibre, whereas ice creams made with C (control) and B1 (ice creams made from 1% peach pulp fibre) was the highest scored by the panellists.

**Conclusions:**

Peach fibre concentrates might be used as a good source of nutraceutical ingredients.

Peach (*Prunus persica* L.) fruits have high economic and nutritional value. Carbohydrates, organic acids, minerals, and dietary fibre (DF) are among the major components of peach fruit, which contribute to the nutritional quality of both fruits and juices (1, 2). Peach fruits have aperient properties, are appropriate to prevent costiveness, and are used for the treatment of duodenum ulcers. Phenolic acids, flavonoids, and anthocyanin compounds serve as major sources of potential antioxidants in peach fruit, which might be responsible for these therapeutic functions (2). Peaches are a tropical fruit, which means that the ripening process is triggered and driven by a plant hormone called ethylene. This is responsible for its short shelf-life and represents a serious constraint for its efficient handling and transportation (3, 4).

DF can provide a multitude of functional properties when they are incorporated into food systems (5). But, there are little data dealing with the study of the functionality of DF in ice creams (6). To the best of our knowledge, no data have been published regarding the effect of drying technique on physicochemical characteristics of fibre obtained from peach or about possible differences on physical properties and functionality of peach fibre because of the type of tissue used (peel or pulp) (7). Fibre addition contributes to the modification and improvement of the texture, sensory characteristics, and shelf-life of food because of its water-binding capacity, gel-forming ability, and fat mimetic, antisticking, anticlumping, texturising, and thickening effects (6).

Peach fibre is a valuable nutrition component and its non-use for human diet constitutes a real nutritional loss because peach fibres contain extractable bioactive compounds which can be used as value-added materials. The objective of the present study is to evaluate the functional properties of fibres obtained from peach peel and pulp for possible enrichment of the quality and nutritional contents of ice creams.

## Materials and methods

### Materials

Cow's milk and cream were purchased from the Research and Application Farm of Atatürk University, Erzurum, Turkey. The peach fruits were purchased from a supermarket in Erzurum, Turkey, and classified visually for colour and physical damage. Sugar, salep, and emulsifier (mono- and diglycerides) were obtained from the local market. Skimmed milk powder was purchased from a commercial company (Pınar Dairy Products Co., Istanbul, Turkey).

### Preparation of peach peel and pulp fibre

The total weight of the peaches used was 4 kg, and the average diameter of each peach was 7 cm. Pulp and peel were stripped off after each peach fruit was dipped in water and washed. Pulp and peel were submitted to a juice extraction using a domestic appliance with the purpose of eliminating a great part of the water content and its solubility. The remaining solids were contacted with ethanol (96%, v/v) and stirred (600 rpm) for 15 min. Finally, ethanol was discarded and a portion of each remnant (pulp remnant or peel remnant) was dried under 30°C forced air convection for 7 h. As a result of the process, two types of flour were produced: type A flour, obtained from peach peel, and B flour, obtained from peach pulp. The two types of flour were milled in a Mill Laboratory at 2,890 rpm and then at 5,000 rpm until they could pass through a 0.2-mm sieve to recover the peach fibre concentrates and stored at −18°C for subsequent analyses and incorporation studies (7).

### Flour yield

Yield was calculated by dividing the amount of flour produced by the amount of peach used, and the results were converted to g/kg (g of flour per kg of peach).

### Chemical and physical composition of DFs

Moisture content was measured according to the Association of Official Analytical Chemists (AOAC) method (8). Ash was analysed by combusting the sample in a muffle furnace at 550°C for 4 h. The residue was dissolved in HNO_3_ and the mineral constituents (Ca, K, Na, Mg, P, and Fe) were determined using an inductively coupled plasma optical emission spectrophotometer (Optima 2100 DV, ICP/OES, Perkin-Elmer, Shelton, CT). The Kjeldahl method was used to evaluate the protein content (9). The flour's pH was determined using a pH meter (Mettler-Toledo AG 8603, Schwerzenbach, Switzerland) (10). The colour of the flour was monitored using a Minolta colorimeter (Chroma Meter CR-200, Osaka, Japan). DF, that also accounts for structural variability and associated health benefits, has recently been defined by CODEX Alimentarius to be ‘carbohydrate polymers’, which have been obtained from food raw material by physical, enzymatic, or chemical means and which have been shown to have a physiological effect of benefit to health as demonstrated by generally accepted scientific evidence to competent authorities (11, 12). Insoluble dietary fibres (IDFs) and soluble dietary fibres (SDFs) were assessed using the method of Prosky et al. (13). In this method, samples were enzymatically digested under the same conditions as used in the AOAC official method, and the total dietary fibre (TDF) was calculated as the sum of IDFs and SDFs. Total phenolic content was determined for peach peel and pulp flour according to Bunzel et al. (14). The total phenolic contents were evaluated using the Folin–Ciocalteu method (15).

### Water- and oil-holding capacity

A volume of 25 mL of distilled water or commercial olive oil was mixed with 1 g of dry sample. The mixture tubes were centrifuged at 3,000*g* for 20 min and then the supernatant was poured out. After that, the tubes were drained for 10 min by putting them at an angle of 45°. The residue was weighed and water-holding capacity (WHC, g of water per 100 g of sample) and oil-holding capacity (OHC, g of oil per 100 g of sample) were calculated according to Gould et al. (16) and Caprez et al. (17), respectively.

### Manufacture of ice creams

The ice cream samples were prepared at the Seref patisserie, Erzurum, Turkey. First, the fat content of cow's milk was adjusted to 6% by adding cream, and the prepared milk was separated into five equal amounts of 2 kg each. Skimmed milk powder (125 g), sugar (405 g), a stabiliser (salep; 16.2 g), and emulsifiers (mono- and diglycerides; 6.75 g) were added to these milk samples. Flour samples obtained both from peach peel and pulp were also added to the milk samples (65°C) at two different mass fractions (1 and 2%). The mixtures were subjected to pasteurisation at 85°C for 25 min and stored at 4°C for 24 h, after which they were placed in an ice cream machine to freeze and then hardened at −22°C for 24 h. They remained at −18°C during all physical, chemical, mineral, and sensory analyses.

### Ice cream analysis

Dry matter, fat and ash contents, acidity (°SH), and pH of ice cream samples were determined using the method of Demirci and Gündüz (18). Mineral contents (Ca, K, Na, P, S, Mg, Fe, Mn, Zn, and Ni) of ice cream samples were described by Guler (19). Overrun was detected by the method proposed by Jimenez et al. (20) and calculated using the following equation:

$$\begin{array}{l} \text{Overrum}=[V(\text{ice cream})-V(\text{mix})/V(\text{mix})]\times 100\\ \left(V:\text{volume}\right) \end{array}$$

Time period (sec) from the initial dripping to complete melting was determined using the method of Güven and Karaca (21). Samples of 25 g were heated to melt at room temperature (20°C) by putting them in a beaker capped with a 0.2-cm wire mesh screen. Initial dripping and complete melting times of the samples were calculated in seconds. Viscosity of the ice cream samples was measured at 4°C using a digital Brookfield viscometer (22).

The colour parameters of ice cream samples were obtained by measuring L* (brightness, 0: black, 100: white), a* (+: red, −: green), and b* (+: yellow, −: blue) values using the method of described in (23).

### Sensory assessment

Eight professional panellists participated in the study and evaluated the ice cream samples using a score test for flavour, body and texture, colour and appearance, resistance to melting, and general acceptability. Hardened ice cream samples were tested at a serving temperature of −10°C and given scores for their sensory characteristics using a scale ranging from 1 (poor) to 9 (excellent). All panellists were preferred to be non-smokers (24).

### Statistical analysis

In total, five experimental groups were formed: two types (from peach peel and pulp) of flour, two fibre concentrations (1 and 2%), and a control group without fibres. Statistical analysis was carried out using SPSS 15.0 (SPSS, Inc., Chicago, IL) (25) software. Data were subjected to a multiple analysis of variance, and the average values were compared using Duncan's multiple range test.

## Results and discussion

The mean yield of peach peel and peach pulp flour was calculated to be 57.1 and 100.5 g/kg, respectively. Pulp was found to give a larger amount of flour than peel.

### Chemical and physical characteristics of DFs

Chemical compositions of fibre samples produced from peach peel and pulp are given in Table 1. Peach pulp flour was found to have higher moisture content (11.36%) than peach peel flour (9.21%). Abdul Aziz et al. (26) stated that mango pulp flours have consistently higher moisture content than mango peel flours, and moisture content increased in the pulp as the ripening process progressed and are in agreement with our report.

**Table 1:** The chemical and physical properties of peach peel and pulp flour

	Peach peel (type A)	Peach pulp (type B)
Chemical analysis		
Moisture (g/100 g)	9.21±0.50	11.36±0.20
Ash (g/100 g)	4.3±0.28	4.11±0.37
pH	4.21±0.43	3.58±0.64
Protein (g/100 g)	7.51±0.71	6.90±0.20
Total phenolic content^c^	1002±1.91	1566±0.91
Total dietary fibre (g/100 g)	57.48±0.25	51.05±0.24
Insoluble dietary fibre (g/100 g)	53.15±0.41	46.21±0.63
Soluble dietary fibre (g/100 g)	12.00±1.10	8.53±0.72
Minerals (mg/kg)		
Ca	88.36±0.18	53.60±0.37
K	1269±0.83	1744±0.03
Mg	97.72±0.15	55.30±0.43
Zn	0.54±0.27	0.27±0.52
Fe	7.14±0.29	2.43±0.50
Physical analysis		
L*	58±1.00	79.7±0.06
a*	11.42±0.17	6.99±0.10
b*	37.49±0.71	28.71±0.46
WHC^a^	13.10±0.92	21.87±0.70
OHC^b^	2.88±0.64	1.76±0.32

L*, lightness; a*, redness (+); b*, blueness (−).

a Water-holding capacity (g water per g sample).

b Oil-holding capacity (g oil per g sample).

c mg/100 g of fibre concentrates.

As observed in Table 1, the ash content of peach peel and pulp flour was found to be 4.3 and 4.11%, respectively. Rodríquez-Ambriz et al. (27) found that banana flour had an ash content of 4.4%, whereas Juarez-Garcia et al. (28) found this rate to be 4.7%. These findings were in agreement with the findings of the present study.

The WHC value obtained for the peach peel flour was lower than that obtained for the peach pulp flour, and that found (21.87 g water per g dry sample) in the present study was lower than that reported by Grigelmo-Miguel and Martín-Belloso (29) in peach DF (between 9.2 and 12.1 g of water per g of dry sample). de Escalada Pla et al. (7) found that the WHC value of peach pulp fibre was 24 g water per g dry sample, whereas the WHC value of peach peel fibre was 25 g water per g dry sample. OHC is among the important functional properties of peach flour and was found in the peach peel (2.88 g oil per g dry sample) and pulp flour samples (1.76 g oil per g dry sample) in our study. When compared with the results of previous studies, the peach pulp flour values were lower than the value of 2.2 g of oil per g of dry sample found in the study of Rodríquez-Ambriz et al. (27). It was stated by de Escalada Pla et al. (7) that the OHC value in peach pulp fibre was 1.81 g oil per g dry sample and in peach peel fibre was 2.03 g oil per g dry sample. Calvache et al. (30) reported high values of OHC in DF from peach bagasse *P. persica* L., which is in agreement with the results obtained in this study.

The mean L*, a*, and b* values were found to be 58, 11.42, and 37.49 in peach peel flour, and 79.7, 6.99, and 28.71 in peach pulp flour, respectively. It was found by monitoring the samples in the present study that the flour obtained from peach peel had a darker colour than that produced from pulp. Because of the possible existence of some browning contributor enzymes, such as polyphenol oxidase, and the occurrence of the Maillard reaction (31), a significant colour change was observed during the peel drying process in the present study, from which dark brown powder was produced from the peel. The L*, a*, and b* values were found to be 76, 5.5, and 29.39 in peach pulp flour, and 56, 12.2, and 34 in peach peel flour, respectively (7), and these results are in agreement with the results of the present study.

Antioxidants play an important role in the prevention of oxidative stress-related diseases (32). Quantitatively, the main dietary antioxidants are polyphenols, followed by vitamins, and carotenoids (33). Goñi et al. (34) stated that polyphenols associated with polysaccharides and proteins in cell walls are significant constituents of DF. Table 1 shows the polyphenol contents of the peach peel and pulp flour types. The pulp flour (1,566 mg/100 g) was found to have the highest content of polyphenols. The total phenolics of the pulp and peel of three different peach fruit varieties (Golden, Shireen, and Shahpasand) was determined by Manzoor et al. (2). Among the different peach varieties tested, the peel and pulp of cv. Golden exhibited the highest phenolic contents (1,354.5 and 881.3 mg gallic acid equivalents (GAE) per 100 of DW, respectively). The findings given above are in accordance with those found in the present study.

As observed in Table 1, peach peel fibres are rich in protein (7.51 g/100 g). Grigelmo-Miguel and Martín-Belloso (29) reported that the protein content (5.44–6.29%) of peach fibre concentrate was also low and that it was the only component, among those studied, that decreased throughout the peach harvest time. Ahmed et al. (35) reported that the sodium content was significantly lower than other minerals (55–86 mg/kg); however, the fibres were rich in potassium.

TDF content in the peach peel flour was found to be 57.48% (Table 1), the majority of which was represented by IDF (53.15%) and the rest by SDF (12.00%). Compared with this finding, in a fibre-rich fraction of chia (*Salvia hispanica*), Alfredo et al. (36) reported TDF, IDF, and SDF contents to be 56.46, 53.45, and 3.01 g per 100 g respectively, whereas in orange peel were found, determined by Chau and Huang (37), to be 57, 47.6, and 9.4 g per 100 g, respectively. These rates were 64.1, 55.2, and 8.9 g per 100 g, respectively, found by Ruales and Zumba (38) in guava and were 66.8, 58.6, and 8.2 g per 100 g, respectively, found by Yangılar (39) in green banana flour. The findings given above are in agreement with those found in the present study.

### Physical and chemical characteristics of ice cream samples

The results of some physical and chemical analyses and mineral contents of ice cream samples are given in Tables 2 and 3. The dry matter content of the control sample was lower than that of other samples at significant levels (*p*<0.05). The dry matter rate of ice creams increased with the addition of peach fibre. As observed in Table 2, the addition of DF significantly affected the fat and acidity values (*p*<0.05). The pH values of ice cream samples ranged from 5.89 to 6.51 in samples with fibre.

**Table 2:** Some chemical and physical properties of ice cream samples with peach fibre

Analysis	C	A1	A2	B1	B2
Dry matter (%)	40.01±0.01^a^	40.45±0.04^a^	41.21±0.16^b^	40.33±0.30^a^	41.05±0.05^b^
Ash (%)	0.98±0.00^a^	1.01±0.02^ab^	1.14±0.03^d^	1.07±0.02^bc^	1.11±0.02^cd^
Fat (%)	6.55±0.07^b^	6.11±0.01^a^	5.98±0.02^a^	6.00±0.03^a^	6.06±0.08^a^
Acidity (%)	0.30±0.00^ab^	0.31±0.02^c^	0.34±0.03^d^	0.28±0.01^a^	0.30±0.00^ab^
pH	6.51±0.02^c^	6.10±0.14^b^	6.15±0.04^b^	6.04±0.04^ab^	5.89±0.04^a^
L*	87.17±0.24^e^	78.18±0.28^b^	74.85±0.37^a^	83.87±0.98^d^	80.30±0.43^c^
a*	3.26±0.09^a^	4.02±0.03^bc^	4.13±0.01^c^	3.81±0.10^b^	4.00±0.00^bc^
b*	9.69±0.05^a^	10.84±0.09^c^	11.07±0.21^d^	10.12±0.17^ab^	10.55±0.57^bc^

* Mean values followed by different letters in the same row are significantly different (*p*<0.05).

C, control without fibre; A1, ice cream made with 1% (w/w) peach peel fibre; A2, ice cream made with 2% (w/w) peach peel fibre; B1, ice cream made with 1% (w/w) peach pulp fibre; B2, ice cream made with 2% (w/w) peach pulp fibre.

**Table 3:** Elemental composition (mg/kg) of the ash in ice creams with peach fibre

Concentrations of minerals	C	A1	A2	B1	B2
Ca	1981.11±7.96^a^	2157±26.82^c^	2256±8.57^d^	2101±2.18^b^	2104±5.65^b^
K	2050±14.14^a^	2174.05±25.18^b^	2435.33±49.96^e^	2263±36.76^c^	2372.82±32.97^d^
Na	488.30±3.81^d^	463.24±4.58^c^	438.78±0.82^b^	413.96±5.60^a^	459.49±2.24^c^
P	1038.45±9.12^a^	2224.21±7.19^b^	2342.78±37.85^c^	2518.21±36.18^d^	2618.67±29.25^e^
S	541.32±1.87^c^	628.60±2.12^e^	590.18±1.61^d^	494.32±4.70^a^	513.56±4.73^b^
Mg	124.65±0.49^a^	161.45±1.47^b^	188.01±2.80^d^	179.35±0.91^c^	192.85±2.04^d^
Fe	10.47±0.16^a^	12.18±0.26^b^	12.58±0.18^c^	11.01±0.04^a^	14.43±1.72^d^
Mn	0.46±0.01^a^	0.54±0.03^b^	0.69±0.01^c^	0.55±0.05^ab^	0.66±0.04^bc^
Zn	73.54±0.65^a^	99.85±0.21^b^	112.21±3.12^d^	100.52±0.29^b^	106.77±3.50^c^
Ni	1.59±0.01^a^	1.60±0.02^a^	1.69±0.02^c^	1.61±0.04^b^	1.70±0.01^c^

*Mean values followed by different letters in the same row are significantly different (*p*<0.05). C, control without fibre; A1, ice cream made with 1% (w/w) peach peel fibre; A2, ice cream made with 2% (w/w) peach peel fibre; B1, ice cream made with 1% (w/w) peach pulp fibre; B2, ice cream made with 2% (w/w) peach pulp fibre.

**Fig. 1:**
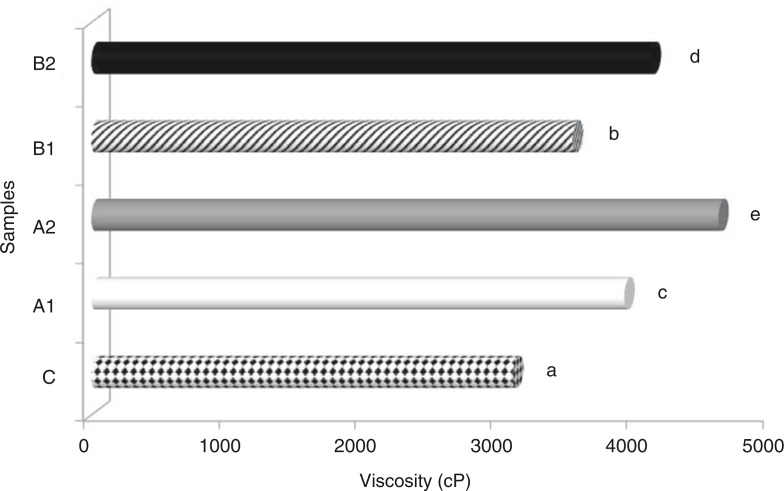
Viscosity values (20 rpm) of ice cream mixes produced using peach fibre. Different letters above the bars indicate significant differences by Duncan multiple comparison test (*p*<0.05).

Viscosity, accepted to be among the significant characteristics of an ice cream mixture, may result in good body and texture properties in the production process of ice cream. From this point of view, it is important to measure viscosity to determine how peach flour may affect the characteristics of an ice cream mixture. As shown in Fig. 1, a lowest viscosity value was obtained in the control sample (3,100 cP) and the highest in sample A (with 2% of peach peel flour added; 4,600 cP). These findings are in agreement with the results of Hwang et al. (40) for ice cream samples with grape wine lees, Dervisoglu and Yazici (5) for ice cream samples with citrus fibre, Cakmakci et al. (41) for ice creams with oleaster (*Elaeagnus angustifolia* L.) flour, and Yangılar (42) for ice cream samples containing date fibre.

The lightness (L*) values of ice cream samples were close to each other, but they were significantly higher for the A1 and A2 samples than for others (Table 2). All of the samples taken into consideration were found to have negative greenness rates, whereas B1 and B2 samples seemed to be similar to and sometimes higher than the others. The colour rates of the samples were affected favourably by an increase in the concentration of peach fibre (*p*<0.05). Samples had negative a* (greenness) values, and the A2 (4.13) sample was significantly higher than the other samples. The b* values were increased by the addition of peach peel fibre. Samples C (9.69) gave the lowest b* rate, whereas the highest rate was received from the A2 (11.07) samples. Dervisoglu and Yazici (5) reported that the addition of citrus fibre increased the colour properties; these results are in agreement with the results of the present study.

**Fig. 2:**
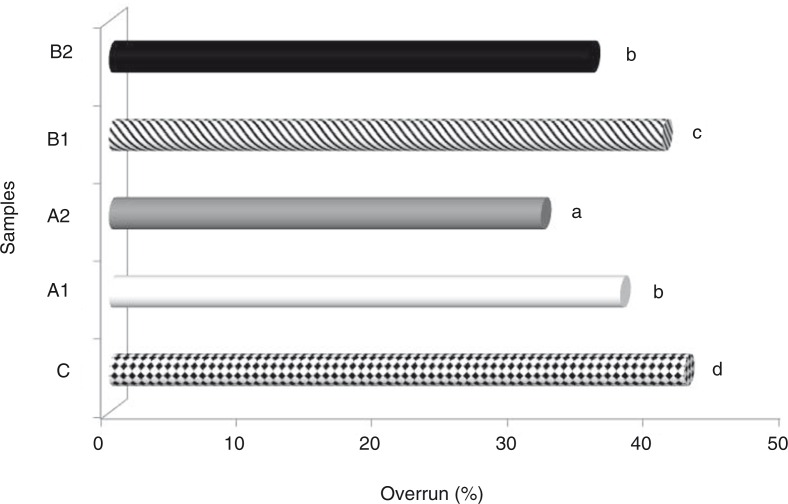
Overrun values of ice cream mixes produced using peach fibre. Different letters above the bars indicate significant differences by Duncan multiple comparison test (*p*<0.05).

Overrun and melting are associated with the volume of air involved in the manufacturing process. This property can shape the structure of the final product because the air present in the ice cream can provide it with a light texture and affect some physical properties, such as melting and hardness (43). All of the ice cream samples in the present study showed much lower overrun values (31.8–42.35%) compared with the values reported in the literature (80–120%). Although the addition of peach fibre lowered the overrun rate of the ice cream samples (*p*>0.05), the control samples showed higher overrun rates than the peach flour samples (Fig. 2). Because the viscosity of the ice cream increased with fibre samples, it was possible that less air was incorporated into the ice cream mixture with fibre during batch freezing, which resulted in a lower overrun compared with the control (without fibre). The results of the study carried out by El-Samahy et al. (44) showed that overrun decreased in ice cream as cactus pulp was added, which could be dependent on the increase in the viscosity of the mixture. Hwang et al. (40) reported that the overrun values of ice cream samples decreased significantly with the addition of grape wine lees. It was found by Sun-Waterhouse et al. (45) that the overrun rate of ice cream containing green kiwifruit was 90.5%, which is higher than that in the present study. Results similar to those found in the present study were found by Cakmakci et al. (41) who studied producing ice cream with oleaster (*E. angustifolia* L.) flour.

**Fig. 3:**
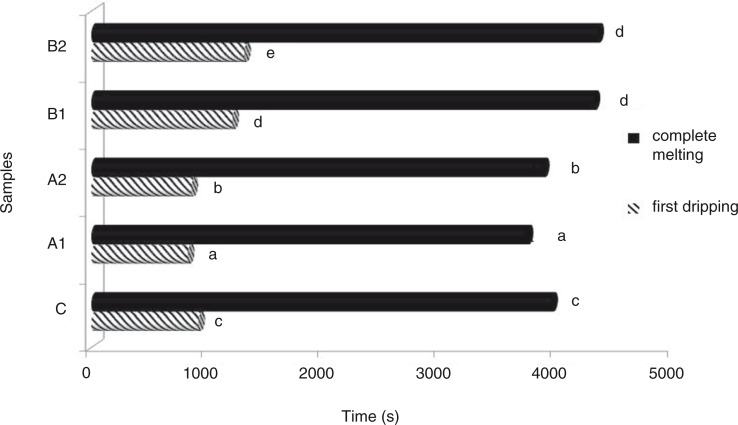
First dripping and complete melting times of ice cream samples with added peach fibre. Different letters above the bars indicate significant differences by Duncan multiple comparison test (*p*<0.05).

As observed in Fig. 3, the length of the time period required for the melting process to be completed was found to be significantly longer in B2 samples with the addition of fibre content. Such a situation may result from the addition of some components with the ability to absorb water in B. The ice cream with B2 revealed the longest complete melting time (0.43 g/min), whereas the shortest complete melting time (0.37 g/min) was shown by the A1 sample. It was stated by Akin et al. (22) that the reason for a decrease in the melting rate of ice cream with added inulin might be because of the ability of inulin to prevent water molecules from moving freely. B (1 and 2%) concentrations affected the initial dripping time positively (Table 2).

The results of the present study indicate that the length of the time period until the initial dripping became prolonged as the fibre content of the ice cream samples increased (*p*<0.05). Dervisoglu and Yazici (5) reported that ice cream samples with citrus fibre had longer dripping times and a similar result was also reported by Yangılar (39) in ice cream with green banana peel flour.

Table 3 shows the changes in the mineral contents of the ice cream samples. The contents of K, Mg, and P in ice cream samples significantly increased, and statistically significant differences were found in terms of the major element contents, such as Ca, K, P, and Mg, between the samples, except for the S concentration in all ice cream samples. The increase in these mineral contents may be because of the high K, Mg, and P concentrations in peach fruit. As observed in Table 3, the K content of peach peel was between 2174.05 and 2435.33 mg/kg, and the A2 sample in the present study revealed the highest K rate. Manzoor et al. (2) reported that mineral analysis results were in agreement with those of Basar (46) who reported that K was the most abundant nutrient in fruits of different peach varieties, followed by Mg, Ca, Fe, Zn, and Mn. The A1 sample gave the highest S rate (628.60 mg/kg), whereas the B1 sample had the lowest (494.32 mg/kg). In our study, the Na values of ice cream were found to be decreased in peach fibre-added samples. If we evaluate the results only in the study, we see that the decrease is seen in the fibre additive rather than in the control; however, the highest increase is seen in the Na rate coming from the peel flour. A similar result was reported by Dağdemir (47) for vegetable marrow (*Cucurbita pepo* L.) added to ice cream. Decreasing Na in the human diet may provide protection from hypertension in people who are sensitive to high levels of Na. A2 revealed the highest Zn content (112.21 mg/kg). It was stated by Wu et al. (48) that Zn could play some vital roles by serving as a non-enzymatic antioxidant and protecting cells from oxidative damage. Even if peach fibre contains small doses of Fe, Zn, Ni, and Mn, which carry the capability of contributing to the antioxidant activity of fruit (49), in the present study, when added, fibre seems to rise significantly the Fe, Zn, and Mn contents of the ice cream samples (*p*<0.05). Similar results were reported for Cape gooseberry (*Physalis peruviana* L.) added to ice cream by Erkaya et al. (50) and for the ice cream samples with date fibre by Yangılar (42).

### Sensory evaluation of ice creams

**Fig. 4:**
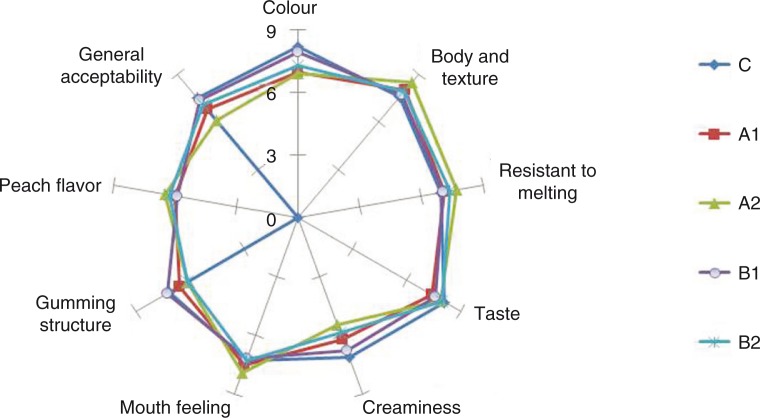
Some sensorial properties of ice cream samples.

The results of the sensory evaluation of the ice cream samples on a scale from 1 (poor) to 9 (excellent) are shown in a radar plot in Fig. 4. Fortifying ice cream with DF had a significant effect on all sensory properties overall. All of the fibre-enriched samples received higher scores for total evaluation in terms of sensory characteristics (*p*<0.05). Colour scores were significantly different, between 6.95 and 8.20. The lowest score was determined for sample A2. Peach fibre gave a slight yellowish colour to ice cream samples. The A1 and A2 samples containing peach peel fibre showed relatively high scores in terms of organoleptic characteristics such as body and texture, resistance to melting, and mouth feeling compared with the control group. The B2 sample had a similar mouth feeling, showed resistance to melting, and gave the same generally acceptable ratings as the control sample. The highest values for general acceptability belonged to the C, B1, B2, A1, and A2 samples. Processing of peach into fibre presents an excellent opportunity for use as a functional ingredient to ensure its extended consumption and reduce wastage.

## Conclusions

The enrichment of ice cream with peach fibre is an effective way to enhance nutritional and physiological aspects by influencing the rheological and thermal properties of the final product. Peach fibre alone or with ice cream stabilisers was successfully used in ice cream production. The addition of peach peel fibre affected moisture, fat, acidity, ash, and viscosity positively; on the contrary, meltdown, colour, and overrun were affected negatively. Given peach fibre's nutritive value and pleasant flavour, it may be used as a suitable source of natural additive in ice cream production to enhance nutritional values. In our study, among the two parts (peel and pulp) analysed, the present results also revealed that peach pulp exhibited higher moisture, phenolic content, and WHC value compared with that of the peel, indicating that removal of peel from such fruits may induce significant nutrient losses. Therefore, the intake of functional foods along with their fibres can be more beneficial to increased nutritional properties of foods such as ice cream. Therefore, our study may provide a base of knowledge for future research.
